# Delphi-Based Consensus to Determine Core Aspects of Post-Hip-Fracture Surgery Rehabilitation Based on the International Classification of Functioning, Disability, and Health

**DOI:** 10.3390/ijerph192315988

**Published:** 2022-11-30

**Authors:** Che-Li Lin, Chun-De Liao, Yu-Hao Lee, Reuben Escorpizo, Tsan-Hon Liou, Shih-Wei Huang

**Affiliations:** 1 Department of Orthopedic Surgery, Shuang Ho Hospital, Taipei Medical University, New Taipei City 23561, Taiwan; 2 Department of Orthopedics, School of Medicine, College of Medicine, Taipei Medical University, Taipei City 11031, Taiwan; 3 Masters Program in Long-Term Care, College of Nursing, Taipei Medical University, Taipei City 110301, Taiwan; 4 Department of Physical Medicine and Rehabilitation, Shuang Ho Hospital, Taipei Medical University, New Taipei City 23561, Taiwan; 5 Department of Physical Medicine and Rehabilitation, School of Medicine, College of Medicine, Taipei Medical University, Taipei City 11031, Taiwan; 6 Department of Rehabilitation and Movement Science, College of Nursing and Health Sciences, University of Vermont, Burlington, VT 05401, USA; 7 Swiss Paraplegic Research, 6207 Nottwil, Switzerland

**Keywords:** frailty, hip fracture, International Classification of Functioning, Disability and Health, core set, rehabilitation

## Abstract

A hip fracture is a major adverse event for older individuals that has extremely high rates of mortality and morbidity, specifically functional decline. Thus, effective post–hip fracture rehabilitation is crucial to enable patients to regain function and improve their quality of life. Most post–hip fracture rehabilitation programs focus only on physical functioning, but rehabilitation goals related to the quality of life, social participation, and environmental issues are also crucial considerations. This study aimed to develop a core set of considerations based on the International Classification of Functioning, Disability, and Health (ICF) for use as a reference in designing comprehensive rehabilitation programs for patients with hip fractures. For this purpose, we recruited 20 experts from related fields working at a university hospital to complete a three-round Delphi-based questionnaire. Before beginning this process, a literature review related to ICF category selection was conducted. Next, a 5-point Likert scale was employed to rate the importance of each proposed category, and Spearman’s rank correlation coefficient and semi-interquartile range indices were analyzed to rate the consensus status. Categories for the ICF core set of considerations for post-hip-fracture rehabilitation were chosen on the basis of a high level of consensus and a mean score of ≥4.5 in the third Delphi-based questionnaire round. After selection, the ICF core set comprised 34 categories, namely 15 for bodily functions, 5 for bodily structures, 13 for activities and participation, and 1 for environmental factors. The proposed post-hip-fracture rehabilitation ICF core set can serve as a reference for developing effective rehabilitation strategies and goal setting by interdisciplinary teams. However, further feasibility evaluation is recommended for individualized rehabilitation program design.

## 1. Introduction

Hip fracture is a major adverse event for older individuals that has extremely high rates of mortality and morbidity, specifically functional decline [1,2]. Further, it has been identified as one of the most prevalent healthcare problems among elderly people [3]. The incidence of hip fracture increases with age; for men and women, it rises from 22.5 and 23.9 per 100,000 people at 50 years old to 630.2 and 1289.3 per 100,000 people at 80 years old, respectively [4]. Despite surgical repair options, patients with hip fractures still exhibit negative outcomes related to mobility and activities of daily living (ADLs) [2,5]. To improve recovery outcomes, studies have investigated predisposing factors related to the risk of mortality, the recovery of one’s walking ability, and predicting the risk of falls [6,7]. For postoperative rehabilitation, an orthogeriatric care model that uses a comprehensive geriatric assessment was developed by an interdisciplinary team; this model was recommended for optimizing recovery [8,9].

Previously, the primary rehabilitation goal after a hip fracture was to minimize a patient’s hospitalization duration through multidisciplinary team intervention and then to continue with home rehabilitation after discharge. A recent study indicated that early discharge followed by geriatric interdisciplinary home rehabilitation resulted in similar recovery regarding independence in ADLs at 3 and 12 months of recovery compared with hospital geriatric care and rehabilitation [10]. In addition, studies have observed that a home rehabilitation intervention can promote independence in ADLs and lead to improved performance of instrumental ADLs in both the short and long term [11,12,13]. However, contrasting results have been obtained in two other studies, which found no significant improvement in self-care or independence in ADLs after home rehabilitation interventions [14,15]. The inconsistency of these findings may be attributable to differences in the goals and intervention types of these rehabilitation programs. Therefore, various possible dimensions of multidisciplinary team rehabilitation should be comprehensively considered.

The World Health Organization developed the International Classification of Functioning, Disability, and Health (ICF) framework to provide a comprehensive and holistic description of the functioning and disability status of individual patients [16]. This framework covers the components of bodily functions, bodily structures, daily activities, and social participation. In this manner, the ICF framework constitutes a disease classification system that can identify health-related problems and conditions. However, the framework comprises more than 1450 categories, making its clinical application difficult [17]. Thus, for clinical application, more concise ICF categories are necessary for specific diseases and their related disability statuses. Most existing post-hip-fracture rehabilitation programs focus on a patient’s physical functions and self-care ability and thus overlook the maintenance of the patient’s quality of life, social participation, mental health, and environmental health, all of which are also crucial rehabilitation goals. Therefore, to achieve the objective of comprehensive rehabilitation goal setting after hip fracture, the development of a concise ICF core set of considerations is crucial. For this reason, this Delphi-based consensus study was conducted to create such a core set for reference in comprehensive rehabilitation program development for patients with hip fractures.

## 2. Materials and Methods

### 2.1. Study Design and ICF Category Selection

The Delphi-based consensus method was applied to determine the optimal ICF core categories for a post-hip-fracture rehabilitation program [18]. To identify possible factors affecting post-hip-fracture rehabilitation, a systemic review of potential factors was conducted. This review was performed by two reviewers who conducted a search using the following keywords: “hip fractures,” “rehabilitation,” “falls,” and “multi-disciplinary”. All relevant articles in English obtained from the search results were selected for further evaluation, and a quality assessment was performed. The selected articles were then reviewed independently by two reviewers (Lin and Liao), who selected multiple hip fracture rehabilitation-related factors. Data extracted from selected studies including data related to hip fracture and rehabilitation factors and rehabilitation strategies were identified. The Jadad scale was used to evaluate the quality of the randomized controlled trial. The scores ranged from 0 to 5 points, and trials with scores of more than 4 points were considered enrolled in this study. The Newcastle-Ottawa Scale was used to assess the quality of prospective cohort studies. The maximum score was 9 points. Studies with scores of more than 5 points were considered to be of adequate methodological quality. When disagreements arose about hip fracture rehabilitation factors, they were resolved by a third reviewer (Huang). Subsequently, these factors were linked to relevant ICF categories. Based on these categories, an ICF core set questionnaire was developed containing the codes for four new categories: Bodily functions (b; 33), bodily structures (s; 10), activities and participation (d; 36), and environmental factors (e; 19). Finally, three rounds of the questionnaire regarding hip fracture rehabilitation were conducted. This study was approved by the Joint Institutional Review Board of Taipei Medical University (N202101010).

### 2.2. ICF Core Set Consensus Process

**Figure 1:**
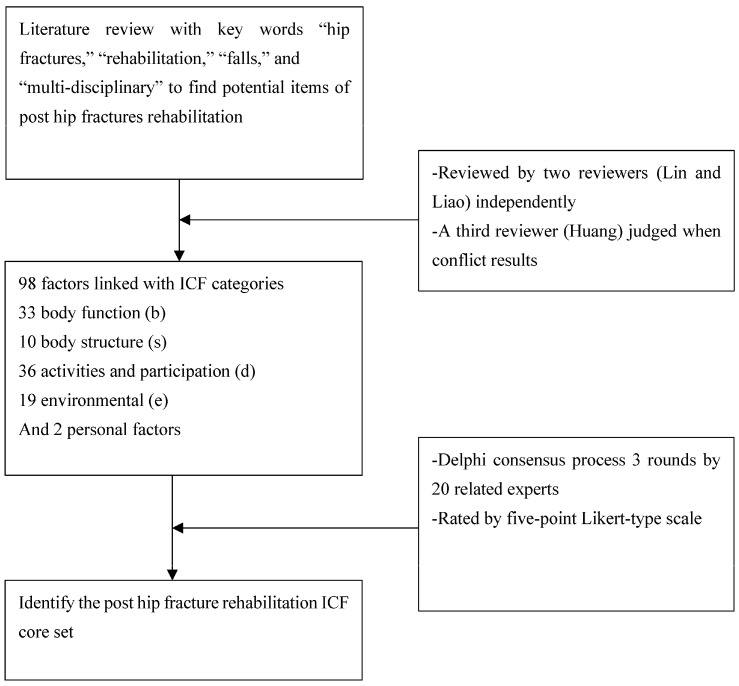
Study flowchart.

The three rounds of the Delphi-based consensus questionnaire survey were conducted between 1 June and 31 August 2022 at a university hospital. Twenty multidisciplinary hip fracture care experts (five physiatrists, three orthopedic surgeons, six physiotherapists, five occupational therapists, and one psychological therapist) were recruited for this hip fracture rehabilitation core set developmental study. All these experts had more than 5 years of clinical experience and the proportion of different experts was based on daily clinical practice hip fracture surgery and rehabilitation by multidisciplinary team intervention in a medical university hospital. These participants were informed of the study objectives, the consensus process methods, and the clinical scenario of hip fracture rehabilitation via email. After the participants had agreed to join this study, the questionnaire was sent to them. The questionnaire contained second-level ICF codes for potential post-hip-fracture rehabilitation-associated categories, and the content of these categories was presented in detail to facilitate the importance rating. The participants rated the importance of each of the selected categories by using a 5-point Likert-type scale (5: Very important; 4: Important; 3: Somewhat important; 2: Not very important; 1: Not important). The questionnaire of three rounds were the same categories for rating scores and each category had an explanation of the content. The scores of each category given by all the participants were averaged, and the scores from the first and second rounds of the questionnaire were used as a reference during the second and third rounds, respectively, to provide information regarding previous scores and enable the participants to re-evaluate their scores for all the items on the basis of their previous scores and those of the other participants. After the three rounds of the questionnaire, the post-hip-fracture rehabilitation ICF core set was developed on the basis of an average Likert scale score of more than 4 points in the final round. The hallmark of this study is illustrated in Figure 1.

### 2.3. Statistical Analysis

To determine the most suitable ICF core set categories for post-hip-fracture rehabilitation, we conducted serial data analysis. Spearman’s rank correlation coefficient (rho) scores were calculated to compare the individual scores of the participants with the mean scores of all the participants for each ICF category in each round of the questionnaire. A rho value of more than 0.7 indicated strong agreement for a category between a participant and all the participants. Regarding the ICF core set, as long as the category scored more than 4.5 on the Likert scale in the third round of the Delphi-based consensus, it was considered suitable for the core set for post-hip-fracture rehabilitation. Data analyses were performed using SPSS (version 17.0; IBM, Armonk, NY, USA), and a p-value of less than 0.05 was considered statistically significant.

## 3. Results

As mentioned, 20 experts from relevant fields completed all three rounds of the Delphi-based consensus questionnaire. In the first round, the mean (SD) Spearman’s rho value was 0.524. In the second round, it is 0.660, and it is 0.748 in the third round. The mean (SD) Likert scores of all categories in all three rounds are presented in Table 1, Table 2, Table 3 and Table 4.

**Figure 2:**
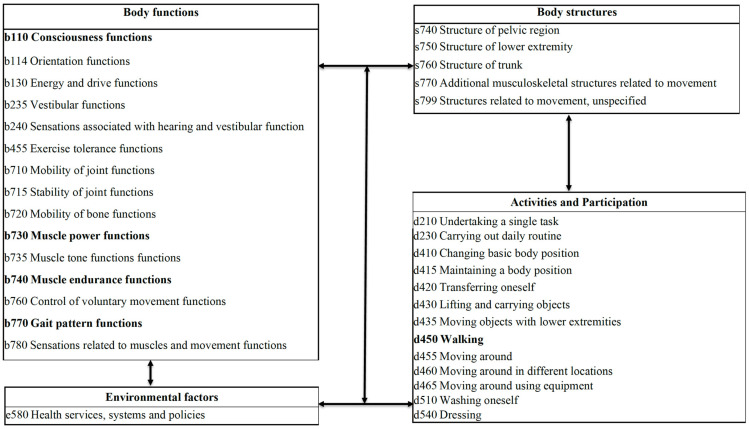
ICF core set of factors associated with post-hip-fracture rehabilitation. The arrows present the association of different categories based on ICF framework.

A total of 34 categories scored more than 4.5 on the Likert scale in the third round of the questionnaire, and thus, these 34 categories were considered suitable for the ICF core set for post-hip-fracture rehabilitation. These categories were divided as follows: 15 for bodily functions, 5 for bodily structures, 13 for activities and participation, and 1 for environmental factors. The categories of Consciousness functions (b110), Muscle power functions (b730), Muscle endurance functions (b740), Gait pattern functions (b770), and Walking (d450) achieved the highest level of expert consensus (5 points on average on the Likert scale; Figure 2).

## 4. Discussion

To describe the functional impairment status of patients with a hip fracture effectively and comprehensively for clinical application, precise ICF category selection for core set formation is essential. This study developed an ICF core set for post-hip-fracture rehabilitation by employing the Delphi consensus process. A total of 34 categories were identified as suitable for the ICF core set; these identified categories can provide multidimensional information for the development of effective rehabilitation programs, which in turn can promote early discharge from the hospital and a return to independence in ADLs after hip fracture. In summary, in addition to focusing on physical activity and strength in the lower limbs, the proposed ICF core set provides a reference for multidisciplinary team rehabilitation program design.

The Consciousness functions (b110) category was considered one of the most crucial categories in the post-hip-fracture rehabilitation core set; this finding is similar to that of a previous study, which noted that 40% of patients with hip fractures also have dementia [19]. This figure indicates the importance of the cognitive function dimension in rehabilitation programs. Recent studies have recommended enhancing rehabilitation strategies by including this dimension to facilitate the recovery of patients with hip fractures and dementia [20,21,22,23]. That is, these studies have suggested enhanced interdisciplinary rehabilitation and care models for hip fracture patients with dementia. Shyu et al. observed long-term benefits from enabling patients to regain their walking ability and physical functions through an interdisciplinary intervention program designed for cognitively impaired older persons after a hip fracture in Taiwan [23]. Cognitive impairment is a key aspect to be considered for effective rehabilitation program design for patients with a hip fracture, and thus, experts in related fields are recommended to recruit cognitive specialists for interdisciplinary rehabilitation program design.

The Muscle power, Endurance, Gait pattern functions, and Walking categories were considered to be among the most crucial categories for inclusion in the ICF core set. These findings are in accordance with those of a previous study, which reviewed multiple hip fracture rehabilitation programs and concluded that the most frequently reported outcomes were associated with ambulation ability [24]. Another study found that postoperative high-frequency physical and occupational therapies in acute settings were related to the recovery of ambulation ability [25]. In addition, the inclusion of the dimensions of bodily functions and activities and participation in our ICF core set demonstrates the importance of ambulation ability and ADLs in rehabilitation goal setting. The bodily functions and activities and participation categories can provide information to facilitate rehabilitation goal-setting by physical and occupational therapists. Further, in addition to muscle strength, motor control, balance, and endurance, energy and drive function training should be incorporated into post-hip-fracture rehabilitation programs. Treadmill gait training, quadriceps training with neuromuscular stimulation, and weight-bearing exercises have been suggested for hip fracture rehabilitation in inpatient settings [26,27,28], whereas progressive resistance training and aerobic, strength, and functional training have been recommended for the improvement of ambulation function in outpatient settings [29,30]. The ICF core set proposed in the present study can provide information to facilitate goal setting for hip fracture rehabilitation by experts as part of an interdisciplinary team intervention.

Based on our ICF core set, the rehabilitation strategy can be focused on these categories. These categories can be included by different experts via a multidisciplinary team intervention. In the aspect of body functions, physiotherapists and occupational therapists can design the rehabilitation program via these core set categories. Similarly, the assessment of body structures can focus on the related dimension of ICF core set by clinical physicians and rehabilitation-related medical staff. The goal setting activities and participation in these ICF core set categories can be applied for functional achievement after the rehabilitation program.

In addition to the bodily functions and activities and participation categories, the bodily structures and environmental factors categories were also considered necessary as ICF core set categories. In addition to the hip region, other bodily structures related to movement were also included in the ICF core set, including those vital for maintaining balance and stabilization. In addition, health services, systems, and policies were considered environmental factors, indicating that post-hip-fracture rehabilitation policies and healthcare resources are crucial for effective functional restoration after a hip fracture. Based on the ICF core set, the healthcare system of the inpatient, post-acute care, and community interact with the functional restoration goals and directions. The ICF core set could provide information for effective healthcare resource use by the government and lessen the economic burden of hip fracture patients.

This study also investigated the effectiveness of the post-hip-fracture rehabilitation ICF core set for comprehensive rehabilitation program design. Under the framework of the ICF, the proposed core set provides categories related to bodily functions, bodily structures, activities and participation, and environmental factors. On the basis of these categories, related experts can set goals for rehabilitation and intervention. Nevertheless, some limitations of the present study should be noted. First, perceptions of post-hip-fracture rehabilitation program designs based on the proposed ICF core set may differ among patients. In addition, experts (e.g., orthopedic physicians) may focus on inpatient intervention for patients with hip fractures. To prevent this problem from occurring, the scenario of considering the rehabilitation status of a patient with a hip fracture was described in the questionnaire. Our goal for the proposed ICF core set is to enable patients with hip fractures to regain their preinjury level of functioning. Clear rehabilitation goals eliminate the problem of subjective interpretations of questionnaire ratings. Second, the experts who participated in this study were from multiple fields. The percentage of each field’s representation was based on our clinical experience of post-hip-fracture rehabilitation; however, other percentage divisions may be more appropriate in other countries or under other healthcare systems; for example, this study recruited no nurses or social workers, which is not to say that the inclusion of such professionals would not be beneficial in other contexts. Finally, whether the feasibility and validity of the proposed ICF core set for patients with hip fractures are applicable to other healthcare systems in other countries or whether experts in those different settings would yield different clinical feasibility ratings for the proposed core set are open questions. Thus, regarding clinical applications, further investigation into the clinical feasibility of the proposed ICF core set is recommended.

## 5. Conclusions

To enable patients with hip fractures to regain their preinjury levels of physical functioning and minimize the socioeconomic burden of care, an effective post-hip-fracture rehabilitation strategy design is crucial, particularly for elderly people. The proposed ICF core set provides a multidimensional framework for rehabilitation program design. The categories of this core set can be considered rehabilitative components that are teachable to patients by experts from related fields and other medical professionals. Furthermore, the proposed ICF core set provides information regarding effective rehabilitation strategies, multidisciplinary team interventions, and goal setting for post-hip-fracture rehabilitation programs. Developing an individualized rehabilitation program under the framework of this ICF core set could be highly beneficial for patients with hip fractures.

## Figures and Tables

**Table 1:** Delphi Consensus rating scores of ICF body function categories for hip fracture rehabilitation.

ICF Code	ICF Body Functions Category Title	Round 1	Round 2	Round 3
b110	Consciousness functions	4.6 ± 0.6	4.9 ± 0.4	5.0 ± 0.2
b114	Orientation functions	4.5 ± 0.6	4.5 ± 0.5	4.7 ± 0.5
b130	Energy and drive functions	4.5 ± 0.6	4.5 ± 0.7	4.5 ± 0.6
b134	Sleep functions	3.8 ± 0.8	3.6 ± 0.8	3.6 ± 0.7
b140	Attention functions	3.8 ± 0.7	4.0 ± 0.6	4.0 ± 0.6
b152	Emotional functions	3.6 ± 0.7	3.6 ± 0.9	3.5 ± 0.8
b164	Higher-level cognitive functions	4.0 ± 0.6	4.2 ± 0.6	4.1 ± 0.5
b176	Mental functions of sequencing complex movement	3.8 ± 0.7	4.0 ± 0.7	4.2 ± 0.6
b210	Seeing functions	3.9 ± 0.9	4.0 ± 0.6	4.0 ± 0.6
b235	Vestibular functions	4.4 ± 0.7	4.5 ± 0.6	4.5 ± 0.7
b240	Sensations associated with hearing and vestibular function	4.3 ± 1.0	4.5 ± 0.5	4.6 ± 0.6
b270	Sensory functions related to temperature and other stimuli	3.6 ± 1.1	3.7 ± 0.7	3.4 ± 0.7
b280	Sensation of pain	4.1 ± 0.8	4.3 ± 0.6	4.2 ± 0.6
b420	Blood pressure functions	3.7 ± 0.9	3.8 ± 0.8	3.7 ± 0.7
b440	Respiration functions	3.8 ± 1.0	3.7 ± 0.7	3.6 ± 0.7
b455	Exercise tolerance functions	4.1 ± 0.6	4.3 ± 0.6	4.5 ± 0.6
b460	Sensations associated with cardiovascular and respiratory functions	3.7 ± 0.8	3.8 ± 0.8	3.8 ± 0.6
b610	Urinary excretory functions	3.1 ± 1.1	3.2 ± 0.9	3.1 ± 0.8
b620	Urination functions	3.1 ± 1.0	3.4 ± 0.9	3.1 ± 0.6
b630	Sensations associated with urinary functions	3.2 ± 1.2	3.3 ± 0.8	3.1 ± 0.8
b710	Mobility of joint functions	4.7 ± 0.6	4.9 ± 0.4	4.9 ± 0.3
b715	Stability of joint functions	4.5 ± 0.6	4.9 ± 0.3	4.9 ± 0.3
b720	Mobility of bone functions	4.6 ± 0.6	4.6 ± 0.6	4.7 ± 0.5
b730	Muscle power functions	4.8 ± 0.4	4.9 ± 0.3	5.0 ± 0.2
b735	Muscle tone functions	4.5 ± 0.6	4.8 ± 0.4	4.9 ± 0.3
b740	Muscle endurance functions	4.7 ± 0.5	4.9 ± 0.4	5.0 ± 0.2
b750	Motor reflex functions	4.1 ± 0.6	4.1 ± 0.6	4.2 ± 0.6
b755	Involuntary movement reaction functions	4.3 ± 0.6	4.2 ± 0.6	4.2 ± 0.6
b760	Control of voluntary movement functions	4.8 ± 0.4	4.7 ± 0.5	4.7 ± 0.6
b765	Involuntary movement functions	4.2 ± 0.7	4.2 ± 0.6	4.2 ± 0.6
b770	Gait pattern functions	4.9 ± 0.4	4.9 ± 0.3	5.0 ± 0.2
b780	Sensations related to muscles and movement functions	4.9 ± 0.4	4.9 ± 0.4	4.9 ± 0.4
b810	Protective functions of the skin	3.2 ± 1.1	3.1 ± 1.1	3.1 ± 0.7

**Table 2:** Delphi Consensus rating scores of ICF body structure categories for hip fracture rehabilitation.

ICF Code	ICF Body Structures Category Title	Round 1	Round 2	Round 3
s110	Structure of brain	3.7 ± 0.9	4.0 ± 0.7	4.0 ± 0.6
s120	Spinal cord and related structures	3.9 ± 0.9	4.2 ± 0.6	4.3 ± 0.6
s140	Structure of sympathetic nervous system	3.5 ± 0.9	3.7 ± 0.9	3.6 ± 0.8
s150	Structure of parasympathetic nervous system	3.4 ± 0.9	3.5 ± 0.9	3.4 ± 0.7
s730	Structure of upper extremity	3.9 ± 1.0	4.1 ± 0.7	4.1 ± 0.6
s740	Structure of pelvic region	4.4 ± 0.5	4.5 ± 0.6	4.6 ± 0.5
s750	Structure of lower extremity	4.6 ± 0.5	4.7 ± 0.6	4.7 ± 0.5
s760	Structure of trunk	4.3 ± 0.5	4.4 ± 0.6	4.6 ± 0.5
s770	Additional musculoskeletal structures related to movement	4.3 ± 0.6	4.4 ± 0.6	4.6 ± 0.5
s799	Structures related to movement, unspecified	4.2 ± 0.8	4.5 ± 0.6	4.6 ± 0.5

Values are 20 experts’ mean ± standard deviation scores on a 5-point Likert-type scale.

**Table 3:** Delphi Consensus rating scores of ICF activities and participation categories for hip fracture rehabilitation.

ICF Code	ICF Activities and Participation Category Title	Round 1	Round 2	Round 3
d110	Watching	3.7 ± 0.9	3.9 ± 0.8	3.8 ± 0.8
d115	Listening	3.2 ± 0.8	3.5 ± 0.8	3.4 ± 0.7
d120	Other purposeful sensing	3.5 ± 0.8	3.5 ± 0.9	3.5 ± 0.8
d160	Focusing attention	3.9 ± 0.6	4.1 ± 0.6	4.4 ± 0.7
d210	Undertaking a single task	4.4 ± 0.6	4.4 ± 0.5	4.5 ± 0.5
d220	Undertaking multiple tasks	4.1 ± 0.6	4.3 ± 0.6	4.4 ± 0.6
d230	Carrying out daily routine	4.1 ± 0.9	4.2 ± 1.0	4.5 ± 0.6
d240	Handling stress and other psychological demands	3.6 ± 1.1	3.8 ± 1.0	3.5 ± 0.8
d410	Changing basic body position	4.8 ± 0.4	4.8 ± 0.4	4.8 ± 0.4
d415	Maintaining a body position	4.7 ± 0.5	4.8 ± 0.4	4.8 ± 0.4
d420	Transferring oneself	4.7 ± 0.5	4.9 ± 0.3	4.9 ± 0.4
d430	Lifting and carrying objects	4.4 ± 0.6	4.4 ± 0.8	4.5 ± 0.6
d435	Moving objects with lower extremities	4.5 ± 0.6	4.7 ± 0.6	4.9 ± 0.4
d440	Fine hand use	3.8 ± 0.9	3.9 ± 1.0	3.9 ± 0.8
d445	Hand and arm use	3.9 ± 0.9	4.1 ± 0.8	4.0 ± 0.6
d450	Walking	4.6 ± 0.5	4.8 ± 0.4	5.0 ± 0.2
d455	Moving around	4.6 ± 0.5	4.9 ± 0.4	4.9 ± 0.3
d460	Moving around in different locations	4.6 ± 0.5	4.9 ± 0.3	4.9 ± 0.4
d465	Moving around using equipment	4.6 ± 0.6	4.8 ± 0.4	4.7 ± 0.7
d470	Using transportation	4.3 ± 0.6	4.4 ± 0.6	4.2 ± 0.7
d475	Driving	3.8 ± 1.0	3.9 ± 0.8	3.7 ± 0.7
d510	Washing oneself	4.5 ± 0.8	4.7 ± 0.6	4.7 ± 0.5
d520	Caring for body parts	3.9 ± 0.8	4.1 ± 0.9	4.2 ± 0.6
d530	Toileting	4.5 ± 0.8	4.5 ± 0.8	4.4 ± 1.0
d540	Dressing	4.4 ± 0.8	4.5 ± 0.7	4.5 ± 0.6
d550	Eating	3.9 ± 1.2	3.8 ± 1.0	4.0 ± 0.7
d560	Drinking	3.8 ± 1.2	3.7 ± 1.1	3.8 ± 0.9
d620	Acquisition of goods and services	3.9 ± 1.0	3.9 ± 1.0	3.7 ± 0.6
d630	Preparing meals	3.9 ± 0.9	3.9 ± 0.8	3.7 ± 0.5
d640	Doing housework	3.9 ± 0.9	3.9 ± 0.8	3.8 ± 0.6
d660	Assisting others	3.7 ± 1.0	3.9 ± 0.7	3.6 ± 0.6
d770	Intimate relationships	3.5 ± 1.1	3.7 ± 1.0	3.4 ± 0.7
d850	Remunerative employment	3.7 ± 1.0	3.7 ± 1.1	3.5 ± 0.8
d910	Community life	3.8 ± 0.8	4.0 ± 0.8	3.8 ± 0.5
d920	Recreation and leisure	4.1 ± 0.7	4.1 ± 0.7	4.1 ± 0.4
d930	Religion and spirituality	3.8 ± 0.9	3.9 ± 0.9	3.6 ± 0.6

Values are 20 experts’ mean ± standard deviation scores on a 5-point Likert-type scale.

**Table 4:** Delphi Consensus rating scores of ICF environmental factors categories for hip fracture rehabilitation.

ICF Code	ICF Environmental Factors Category Title	Round 1	Round 2	Round 3
e115	Products and technology for personal use in daily living	4.0 ± 0.9	3.9 ± 0.8	3.8 ± 0.7
e120	Products and technology for personal indoor and outdoor mobility and transportation	4.1 ± 0.9	4.1 ± 0.6	4.0 ± 0.6
e135	Products and technology for employment	3.5 ± 1.0	3.5 ± 0.8	3.4 ± 0.7
e150	Design, construction and building products and technology of buildings for public use	4.0 ± 1.0	3.9 ± 0.7	3.7 ± 0.8
e155	Design, construction and building products and technology of buildings for private use	4.0 ± 1.0	4.0 ± 0.7	3.9 ± 0.7
e225	Climate	3.4 ± 1.1	3.3 ± 1.1	3.0 ± 0.7
e240	Light	3.1 ± 1.0	3.2 ± 0.9	3.0 ± 0.8
e310	Immediate family	4.3 ± 0.7	4.3 ± 0.6	4.3 ± 0.7
e315	Extended family	3.6 ± 0.6	3.6 ± 0.8	3.4 ± 0.8
e320	Friends	4.0 ± 0.8	4.1 ± 0.6	4.0 ± 0.6
e325	Acquaintances, peers colleagues, neighbours and community members	3.7 ± 0.7	3.9 ± 0.6	3.9 ± 0.7
e450	Individual attitudes of health professionals	4.0 ± 1.0	4.0 ± 0.8	4.0 ± 0.7
e460	Societal attitudes	3.6 ± 1.2	3.7 ± 0.7	3.6 ± 0.6
e525	Housing services, systems and policies	4.3 ± 0.8	4.2 ± 0.5	4.2 ± 0.6
e540	Transportation services, systems and policies	4.2 ± 0.8	4.3 ± 0.6	4.4 ± 0.6
e570	Social security services, systems and policies	4.0 ± 1.0	4.2 ± 0.5	4.4 ± 0.7
e575	General social support services, systems and policies	4.1 ± 0.9	4.2 ± 0.5	4.3 ± 0.6
e580	Health services, systems and policies	4.4 ± 0.7	4.5 ± 0.6	4.5 ± 0.6
e595	Political services, systems and policies	3.6 ± 1.1	3.7 ± 0.9	3.4 ± 0.7

Values are 20 experts’ mean ± standard deviation scores on a 5-point Likert-type scale.

## Data Availability

The data presented in this study are available on request from the corresponding author.
